# Crystal Structure of Inorganic Pyrophosphatase From *Schistosoma japonicum* Reveals the Mechanism of Chemicals and Substrate Inhibition

**DOI:** 10.3389/fcell.2021.712328

**Published:** 2021-08-11

**Authors:** Qun-Feng Wu, Wei-Si Wang, Shen-Bo Chen, Bin Xu, Yong-Dong Li, Jun-Hu Chen

**Affiliations:** ^1^National Institute of Parasitic Diseases, Chinese Center for Diseases Control and Prevention (Chinese Center for Tropical Diseases Research), Shanghai, China; ^2^NHC Key Laboratory of Parasite and Vector Biology, Shanghai, China; ^3^WHO Collaborating Centre for Tropical Diseases, Shanghai, China; ^4^National Center for International Research on Tropical Diseases, Shanghai, China; ^5^Key Laboratory of Organo-Pharmaceutical Chemistry of Jiangxi Province, Chemistry and Chemical Engineering College, Gannan Normal University, Ganzhou, China; ^6^The School of Global Health, Chinese Center for Tropical Diseases Research, Shanghai Jiao Tong University School of Medicine, Shanghai, China

**Keywords:** *Schistosoma japonicum*, inorganic pyrophosphatase, inorganic pyrophosphate, crystal structure, inhibition

## Abstract

Soluble inorganic pyrophosphatases (PPases) are essential for facilitating the growth and development of organisms, making them attractive functional proteins. To provide insight into the molecular basis of PPases in *Schistosoma japonicum* (*Sj*PPase), we expressed the recombinant *Sj*PPase, analyzed the hydrolysis mechanism of inorganic pyrophosphate (PPi), and measured its activity. Moreover, we solved the crystal structure of *Sj*PPase in complex with orthophosphate (Pi) and performed PPi and methylene diphosphonic acid (MDP) docking into the active site. Our results suggest that the *Sj*PPase possesses PPi hydrolysis activity, and the activity declines with increased MDP or NaF concentration. However, the enzyme shows unexpected substrate inhibition properties. Through PPi metabolic pathway analysis, the physiological action of substrate inhibition might be energy saving, adaptably cytoprotective, and biosynthetic rate regulating. Furthermore, the structure of apo-*Sj*PPase and *Sj*PPase with Pi has been solved at 2.6 and 2.3 Å, respectively. The docking of PPi into the active site of the *Sj*PPase-Pi complex revealed that substrate inhibition might result from blocking Pi exit due to excess PPi in the *Sj*PPase-Pi complex of the catalytic cycle. Our results revealed the structural features of apo-*Sj*PPase and the *Sj*PPase-Pi complex by X-ray crystallography, providing novel insights into the physiological functions of PPase in *S. japonicum* without the PPi transporter and the mechanism of its substrate inhibition.

## Introduction

*Schistosoma japonicum* is one of the major causative agents of schistosomiasis and has a complex life cycle. From egg to adult, *S. japonicum* undergoes one hatch and two parasitic processes. The three steps occur cyclically under natural conditions between *Oncomelania* snails and mammals successively (Han et al., 2009). In the mammalian body, *S. japonicum* develops from schistosomula to adult worms that lay eggs. The developmental stage of *S. japonicum* in mammals is key to accomplishing its life cycle from an immature worm incapable of laying eggs. During the developmental stages of *S. japonicum*, several biological macromolecules are rapidly synthesized in abundance.

The processing of these biosynthetic reactions enables the rapid hydrolysis of inorganic pyrophosphate (PPi, a by-product) by soluble inorganic pyrophosphatase (sPPase), further driving the reactions forward thermodynamically. Hence, sPPase might be significant for facilitating the growth and development of the organism.

Inorganic pyrophosphatase (PPase) is a one-domain globular enzyme containing several divalent metal ions that catalyze the hydrolysis of PPi to two orthophosphates (Pi). The hydrolytic reaction of PPi not only provides a thermodynamic pull for many biosynthetic reactions but also makes Pi available to many biochemical reactions in which inorganic phosphates are involved (Yi et al., 2012). Additionally, PPase is a type of metalloenzyme comprised of two parts—a divalent metal ion and apoenzyme. From a structural point of view, this type of enzyme has a core structure (Benini and Wilson, 2011) and contains a dimer interface and two chemical-binding sites (Sitnik et al., 2003; Sitnik and Avaeva, 2007). Generally, most eukaryotic PPases are homodimers except for the *Trypanosoma brucei brucei* PPase tetramer (Jamwal et al., 2015), whereas prokaryotic PPases are homohexamers (Benini and Wilson, 2011).

Studies on the roles of sPPase are at three different levels: molecular characteristics, functions in cells or protozoa, and metazoa. The properties of sPPase molecules primarily involve enzyme activity (Hoelzle et al., 2010; Stockbridge and Wolfenden, 2011; Costa et al., 2012), functioning in cells or protozoa by regulating the cytosolic concentration of PPi in *Toxoplasma gondii* directly (Pace et al., 2011) and eliminating excess pyrophosphate toxicity in *Saccharomyces cerevisiae* (Serrano-Bueno et al., 2013). Additionally, sPPase is a key polyphosphate metabolism enzyme in *Leishmania* (Espiau et al., 2006). Recent studies have shown that sPPase is necessary for the larval development of some metazoan species (Islam et al., 2003; Ko et al., 2007), such as *Caenorhabditis elegans* and the roundworm *Ascaris*. *S. japonicum* PPase (*Sj*PPase) has shown that the expression quantity from cercaria to adult worm presents a trend from high to low, and expression level in the integument is higher than that in other parts at different stages in the adult (Liu et al., 2006; Chen J. H. et al., 2014). Nevertheless, the functions and molecular basis of *Sj*PPase remain unclear.

This study analyzed substrate PPi metabolism, expressed recombinant *Sj*PPase, and measured the activities under different conditions using colorimetric methods. Furthermore, we revealed the structural features of apo-*Sj*PPase and the *Sj*PPase–Pi complex by X-ray crystallography and provided molecular insights into substrate inhibition of *Sj*PPase through docking experiments.

## Materials and Methods

### Sequence Analysis

Amino acid analysis and molecular weight (MW) and electronic point (pI) determination of protein were performed using BioXM 2.6. Multiple sequence alignment was performed using MUSCLE^1^ and visualized online using ESPript 3.0^2^ (Edgar, 2004; Robert and Gouet, 2014). Protein sequences were primarily selected from pathogens, yeast, and humans. The accession numbers for these sequences were 4QLZ and 4QMB for *S. japonicum*, EAX99718.1 for *Trichomonas vaginalis* G3, CCD82372.1 for *S. mansoni*, CCF72873 for *Babesia microti* strain RI, NP_218145.1 for *Mycobacterium tuberculosis* H37Rv, CAB37743.1 for *Helicobacter pylori*, 1IPW for *Escherichia Coli*, 1E9G for *S. cerevisiae*, and AAH01022.3 for *Homo sapiens*.

### PPi Metabolic Pathway Analysis

Inorganic pyrophosphate metabolism was analyzed by enzymatic reaction analysis, a literature search, and sequence alignment. Enzymatic reactions involving PPi were searched using the search term “diphosphate” in the Enzyme Structures Database.^3^ Further confirmation was done “on a one by one basis” to ascertain the participation of PPi in these reactions. Furthermore, based on PPi location at product or substrate positions in the reactions, these catalysts were divided into PPi-generating enzyme and PPi-utilizing enzyme (including PPi hydrolyase). Enzymes involved in PPi metabolism were searched^4^ and investigated in humans and *S. japonicum*. Furthermore, PPi transporter and member-integrated PPi metabolism-related enzymes were investigated using a literature survey based on the PPi homeostasis model (Villa-Bellosta et al., 2013). Thereafter, a comparison of the PPi metabolic pathway between blood flukes and human hosts was performed using a sequence alignment search of genes and proteins related to PPi metabolism. To search for progressive ankylosis protein homology (ANKH) in *Schistosoma*, the query sequences (Supplementary File 1) were downloaded from UniProtKB. The search term “ANKH family” was used in the “Family and Domains > Protein family” field Databases and included *S. haematobium*, *S. japonicum*, and *S. mansoni* genomes.

### Cloning, Expression, and Purification of Recombinant *Sj*PPase and *Sc*PPase

A forward primer (5′-AATGGGTCGCGGATCCATGTCGGT TGAACGTGGG-3′) and a reverse primer (5′-GGTGGTGGTGCTCGAGTTAAATATTCGTATTACAAAAAT GCC-3′) were used to amplify the *Sj*PPase gene with cDNA as the template. The 25-μl reaction mixture comprised of 2× Taq PCR MasterMix (12.5 μl), 10 μM forward primer (1 μl), 10 μM reverse primer (1 μl), cDNA template (1 μl), and water (9.5 μl). The cycling conditions were set as follows: initial denaturation at 95°C for 5 min, 30 cycles of PCR including denaturation at 95°C for 30 s, annealing at 55°C for 30 s, and extension at 72°C for 1 min. The full-length gene of soluble PPase from *S. japonicum* was inserted by the In-fusion cloning method into plasmid pET-28a (+) between two restriction sites: BamH I and Xho I. The recombinant plasmid (*Sj*PPase-pET-28a) was transformed into *E. coli* BL21 (DE3), and then the bacteria-harboring *Sj*PPase-pET-28a was conserved in LB medium containing 15% glycerol and 50 mg kanamycin per liter at -80°C for protein expression. The product of small-scale expression was analyzed by sodium dodecyl sulfate-polyacrylamide gel electrophoresis (SDS-PAGE) and Western blotting to verify the expression level of the target protein. The prepared bacteria was revived in 50 ml LB medium containing kanamycin at 37°C, and shaken at 165 rpm for 7 h. The suspension was then decanted into 1 L of LB culture, incubated at 37°C, and shaken at 165 rpm. When OD_600_ approached 1.4, 0.7 mM isopropyl-β-d-thiogalactoside (IPTG) was added to the culture; shaking speed and temperature were altered and set at 195 rpm and 24°C, respectively. After 9 h, the suspension was harvested by centrifugation at 5,000 rpm and a temperature of 4°C for 8 min. The pellet was re-suspended in nickel-nitrilotriacetic acid (Ni-NTA) column buffer A [300 mM NaCl, 50 mM phosphate buffer (pH 7.4), 10 mM imidazole, and 10% glycerol] containing 5 mM phenylmethylsulfonyl fluoride (PMSF) and lysed by sonication. The lysate was clarified by centrifugation at 13,000 rpm and temperature of 4°C for 30 min, and the supernatant was filtered using a 0.22-μm filter membrane. The flowed fraction was loaded into an affinity chromatography column of Ni-NTA resin at a 3-ml/min flow rate using an ÄKTA purifier 10 (GE Healthcare, Chicago, IL, United States). The resin was washed with Ni-NTA column buffer A until the value of UV approached 0 mU, washed again with a mixture of Ni-NTA column buffer B [300 mM NaCl, 50 mM phosphate buffer (pH 7.4), 500 mM imidazole, and 10% glycerol] and Ni-NTA column buffer A at a volumetric proportion of 1:9 until the value of UV approached 20 mU, then eluted with 20 ml buffer B. The eluted protein was dialyzed overnight against diethyl aminoethyl (DEAE) column buffer A [20 mM Tris-HCl (pH 8.0), 50 mM NaCl, 5 mM EDTA, and 10% glycerol]. The protein solution was loaded into DEAE resin at a 3-ml/min flow rate using ÄKTA purifier 10. The resin was washed with DEAE column buffer A until the value of UV approached 0 mU, then eluted with 20 ml DEAE column buffer B [20 mM Tris-HCl (pH 8.0), 500 mM NaCl, 5 mM EDTA, and 10% glycerol]. Each fraction was performed by SDS-PAGE for purification analysis and molecular mass analysis. The eluted protein was dialyzed overnight against a dialysis buffer [50 mM Tris-HCl (pH 7.4), 100 mM NaCl, and 10% glycerol]. The purification products were analyzed using SDS-PAGE, and the purified protein was concentrated to approximately 18 mg/ml using a microcon-10 kDa centrifugal filter (MilliporeSigma, Burlington, MA, United States). The *S. cerevisiae* PPase gene (ScPPase, 1E9G) was synthesized and further constructed in pET-28a (+). The *Sc*PPase recombinant protein was prepared using the method for r*Sj*PPase.

### Native-PAGE

We prepared eight samples containing 100 μg r*Sj*PPase in 100 μl PBS. First, these were equally separated into two groups in which 5 μl of 1 mM Mg^2+^ was added to one group while the other group was without Mg^2+^. Next, four samples from each group were treated with 5 μl of 1 mM glutathione (GSH), 5,5’-dithiobis(2-nitrobenzoic acid) (DNTB), imidodiphosphate (IDP), and 10 μl PBS for 5 min at room temperature. Thereafter, all samples were centrifuged at 3,000 rpm (4°C) for 2 min, and native-PAGE was performed for 3 h at 240 V in an ice bath.

### Enzyme Assay of Recombinant *Sj*PPase

Inorganic pyrophosphatase activity assay was based on a previously described method for HSP90 ATPase activity (Avila et al., 2006). The effects of some molecules, such as magnesium ions (Mg^2+^), PPi, sodium fluoride (NaF), and methylene diphosphonic acid (MDP), on the catalytic activities of *Sj*PPase, were investigated. The detailed conditions are described in Supplementary Table 1. Each group comprised two reactions, test and control reactions, each of which was made up of a 100-μl reaction system. The *Sj*PPase or *Sc*PPase enzyme and magnesium ions, as well as inorganic pyrophosphate and NaF or MDP, were mixed to a 50-μl volume—VC signifies “various concentrations.” VC_*A*_ represents a series of magnesium ions concentrations, including 500, 250, 100, 50, and 25 μM. Various PPi concentrations represented by VC_*B*_ included 2,000; 1,000; 500; 250; 100; 50; 10; and 5 μM. Various NaF concentrations represented by VC_*C*_ included 1 and 0.5 mM. Various concentrations of MDP represented by VC_*D*_ included 2 and 1 mM. The reaction was incubated for 45 min and monitored every 2.5 min. A fluorescence microplate reader was used for fluorescence detection at an excitation of 560 nm and emission detection at 590 nm. All reactions were processed at 25°C. Protein concentration was tested using a bicinchoninic acid assay (BCA assay) using bovine serum albumin as the standard. The curves were analyzed by nonlinear fitting using the GraphPad Prism software, version 5.0 (GraphPad, San Diego, CA, United States). Data for *Sc*PPase and Mg^2+^ ions concentration-response were generated by nonlinear regression to Michaelis–Menten kinetics. Data for *Sj*PPase PPi concentration-response were analyzed by nonlinear regression to substrate inhibition.

### Crystallization, Data Collection, and Structure Determination

Crystallization screening was performed in 96-well plates with sitting drops consisting of 1 μl protein solution mixed with an equal amount of precipitant. The index^TM^ PEG/Ion Screen^TM^ and the PEG/Ion 2 Screen^TM^ kit were used for screening (Hampton Research, Aliso Viejo, CA, United States). Colorless rectangular rod-like crystals were obtained after 2 weeks from the condition 0.2 M sodium malonate, 20% polyethylene glycol (PEG) 3,350. Crystallization conditions were optimized by fine-tuning the pH value, additive type, and salt and precipitation agent concentration. The final conditions (Supplementary Table 2) suitable for X-ray diffraction were reproduced manually in large volumes by the hanging-drop method in 24-well plates, equilibrating 1 μl protein and 1 μl precipitant against 1 ml precipitant. The protein concentration for crystallization was 16–18 mg/ml.

The crystals were collected at a wavelength of 0.9707 Å using an ADSC Quantum 315r CCD detector on a BL17U1 beamline at Shanghai Synchrotron Radiation Facility (SSRF), China. Data were processed and reduced using the HKL2000 package (Otwinowski and Minor, 1997), and relevant statistics are shown in Table 1.

**TABLE 1:** Summary of *Sj*PPase crystallography data.

	4QLZ	4QMB
Space group	P32	P32
Resolution (Å)	34.89–2.33 (2.41–2.33)	37.88–2.60 (2.70–2.60)
**Cell dimension**		
a,b,c (Å)	76.08, 76.08, 123.41	75.75, 75.75, 122.96
α,β,γ (°)	90, 90, 120	90, 90, 120
Total reflections	33,781	24,093
Uni-reflections	3,172	2,381
Mean I/sigma (I)	34.3(2.1)	32.39 (14.28)
Completeness (%)	98.45 (92.05)	99.69 (99.33)
Wilson B-factor	55.59	48.43
R-work	0.2091 (0.2648)	0.2024 (0.2332)
R-free	0.2606 (0.3376)	0.2631 (0.3586)
Z	2	2
No. atoms	4,677	4,588
protein	4,500	4,500
ligands	18	0
water	159	88
Protein residues	561	561
RMS (bonds)	0.009	0.01
RMS (angles)	1.4	1.47
R. favored (%)	92	90
R. outliers (%)	3.1	4
Clashscore	12.84	16.14
Average B-factor	53.5	56.9
Proteins	53.6	57.1
ligands	54.6	
solvent	50.2	46.3

*Uni-reflections, Unique reflections; Z, number of molecules per asymmetric unit; No. atoms, number of atoms; R. favored (%), Ramachandran favored (%); R. outliers (%), Ramachandran outliers (%).*

The structures of *Sj*PPase-Pi and apo-PPase were solved by the molecular-replacement (MR) method using yeast PPase (PDB code: 1E9G) as an initial search model on Phenix (Adams et al., 2010). Thereafter, refinements were conducted using REFMAC, and the models were checked and rebuilt using COOT (Emsley and Cowtan, 2004). Statistical data for the final rounds of refinement are presented in Table 1. The electron density maps were calculated using Phenix, and the figures were generated using PyMOL software (PyMOL molecular graphics system, version 1.3.1; Schrödinger, Inc, New York, NY, United States). The atomic coordinates and structure factors (code 4QLZ and 4QMB) were deposited in the Protein Data Bank.^5^

### Structural Comparison

A structural comparison of the *Sj*PPase and *Sc*PPase ligand and cofactor binding sites was performed. A series of *Sc*PPase PDBs (1e6a, 1e9g, 1m38, 1wgi, 2ihp, 2ik0, 2ik1, 2ik2, 2ik4, 2ik6, 2ik7, 2ik9, and 117e) and *Sj*PPase 4qlz were used for searching the binding sites of ligands and apo-PPase. Combined with the multi-sequencing alignment results, some identical or varying residues between *Sj*PPase and *Sc*PPase at binding sites were found.

### Docking Simulation

Docking simulations were carried out using the C-DOCKER module (Discovery Studio, version 2.1; Accelrys, San Diego, CA, United States). The X-ray crystal structure of *Sj*PPase was used for docking calculation. All water molecules in the crystal structure were removed, while Co^2+^ (M1 and M2) ions in the active site were kept as part of the protein. The CHARMm-force field was applied for docking. The region within 12 Å of Pi was chosen as the active binding site. Random conformations of PPi and MDP were minimized using CHARMm-based molecular dynamics (1,000 steps), which were then docked into the defined binding site. The other parameters were set as default. The CDOCKING ENERGY scoring function was used to rank binding poses.

## Results

### Protein Information

**FIGURE 1:**
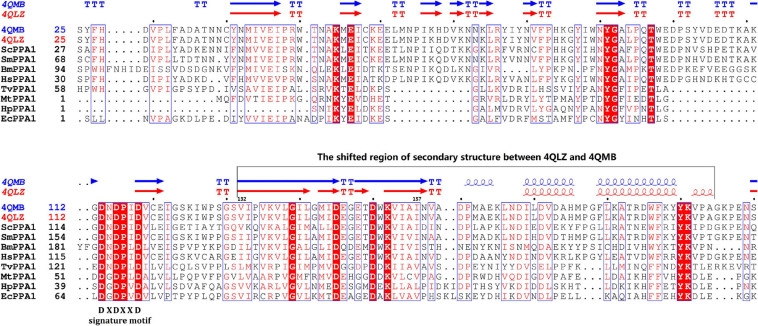
Multisequencing alignment of inorganic pyrophosphatase (PPase) family I. There are 15 conserved residues (in consensus) in the sequences of the PPases. Identical residues are boxed in red. The sequence, DXDXXD, is the signature motif of PPase family I. Secondary structure elements (helices with squiggles, strands with arrows, and turns with TT letters) are displayed for chain A of 4QLZ and 4QMB. Accession numbers for the sequences: *Schistosoma japonicum* (4QLZ and 4QMB), *Trichomonas vaginalis* G3 (EAX99718.1), *S. mansoni* (CCD82372.1), *Babesia microti* strain RI (CCF72873), *Mycobacterium tuberculosis* H37Rv (NP_218145.1), *Helicobacter pylori* (CAB37743.1), *Escherichia Coli* (1IPW), *Saccharomyces cerevisiae* (1E9G), *Homo sapiens* (AAH01022.3). The abbreviation, ScPPA1, represents inorganic pyrophosphatase family I (PPA1) from *S. cerevisiae*. And so on, for other abbreviations.

The 864 bp CDS encoded a 287-aa putative protein with a theoretical molecular weight and isoelectric point (pI) estimated at 32.7 kDa and 6.05, respectively. The protein contained only one pyrophosphatase region. Multiple sequence alignment shows that *Sj*PPase contained the characteristics of a family I PPase, as DXDXXD signature motif and other 12 conserved residues (Figure 1). In addition, the *Sj*PPase protein sequence exhibited 52% identity with *Sc*PPase.

### Comparison of the PPi Metabolism in *Schistosoma* and Human

**FIGURE 2:**
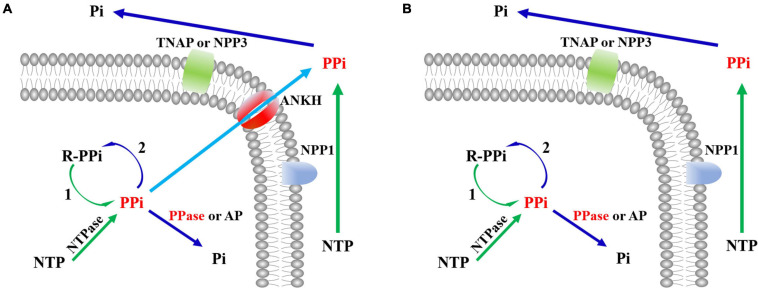
The comparison of PPi metabolism between humans and *Schistosoma*. **(A)** PPi metabolism in humans. **(B)** PPi metabolism in *Schistosoma*. PPi, inorganic pyrophosphate; NTP, nucleotide triphosphates; R-PPi, alkyl and aryl diphosphate; Pi, phosphate; NTases, nucleotidyltransferases; PPase, inorganic pyrophosphatase; NPP1, ectonucleotide pyrophosphatase phosphodiesterase-1; NPP3, ectonucleotide pyrophosphatase phosphodiesterase-3; TNAP, tissue-nonspecific alkaline phosphatase; AP, alkaline phosphatase; ANKH, progressive ankylosis protein; 1, alkyl and aryl transferases; 2, pentosyltransferases and phosphotransferases with an alcohol group as acceptor.

According to enzymatic reaction analysis, intracellular PPi (iPPi) was mainly generated from reactions catalyzed by nucleotidyltransferases, together with alkyl and aryl transferases, while PPi is a by-product. Generated PPi was hydrolyzed by PPase (*EC 3.6.1.1*) or alkaline phosphatase (AP, *EC 3.1.3.1*) or utilized by pentosyltransferases and phosphotransferases with an alcohol group as an acceptor. Based on a literature survey (Villa-Bellosta et al., 2011; Foster et al., 2012), PPi was pumped out from cells by ANKH. Extracellular PPi (ePPi) was hydrolyzed by tissue-nonspecific alkaline phosphatase (TNAP) or ectonucleotide pyrophosphatase phosphodiesterase-3 (NPP3). In addition, a part of ePPi was produced from pyrophosphorolysis of nucleoside triphosphate (NTP) catalyzed by ectonucleotide pyrophosphatase phosphodiesterase-1 (NPP1). All of these formed the general picture of iPPi and ePPi metabolism (Figure 2). We then further searched for protein sequences related to PPi metabolism in *Schistosoma* and humans (Supplementary Table 3). We observed that PPi transporter ANKH exists in the human genome (accession number: NP_473368.1) but was absent in the *Schistosoma* genome. Based on the preceding observations, the comparison of iPPi and ePPi metabolism between humans and *Schistosoma* is shown in Figure 2.

### Expression and Purification of Recombinant *Sj*PPase and *Sc*PPase

**FIGURE 3:**
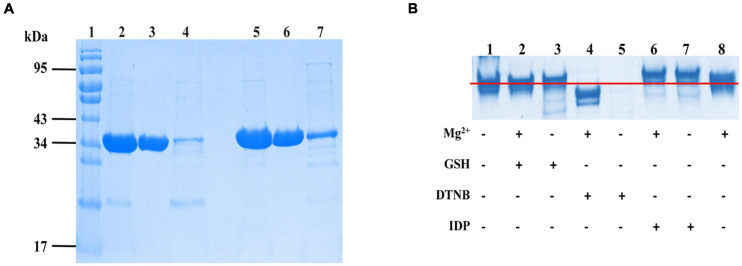
The *Sj*PPases were identified and measured by sodium dodecyl sulfate-polyacrylamide gel electrophoresis (SDS-PAGE). **(A)** The purified results of *Sj*PPase and *Sc*PPase. The samples of *Sj*PPase and *Sc*PPase were loaded in lanes 2–4 and lanes 5–7, respectively. Lane 1, protein ladder; lanes 2 and 5, the fraction eluted by Ni column buffer B; lanes 3 and 6, the flow from the DEAE column; lanes 4 and 7, the elution from DEAE column buffer B; **(B)** Interaction between *Sj*PPase and micromolecules. GSH, glutathione; *DNTB*, 5, 5’-Dithiobis (2-nitrobenzoic acid); IDP, Imidodiphosphate; plus sign (+) represents addition of the left molecule, and minus sign (–) represents non-addition.

The Western blotting results suggested that target proteins were expressed in the supernatant of lysates (data not shown). Protein was purified using a Ni-NTA column followed by the DEAE column. The results (Figure 3A) were displayed by SDS-PAGE using coomassie blue staining.

### Interaction Between *Sj*PPase and Micromolecules

The interaction between PPase and micromolecules was based on the following: the divalent metal-ions as the co-iron of PPase, two substrate affinity sites on the surface of PPase, and cystine residues as the regulatory sites for enzyme activity. A 12% polyacrylamide gel was run for the designed mixtures to improve our understanding of the function of *Sj*PPase micromolecules (Figure 3B). The major components of lanes 1, 2, 3, and 8 were at the same level of electrophoretic distance, while lanes 6 and 7 were at a higher level and lane 4 was at a lower level. The sample for lane 5 was precipitated after pre-treatment. The comparison between the nontreated sample (lane 1) and the sample treated with DNTB (lane 5) revealed that DNTB could destroy the structure of *Sj*PPase. Having observed the differences between lanes 5 and 4 (samples treated with DNTB and Mg^2+^, respectively), this study shows that Mg^2+^ could stabilize the *Sj*PPase structure. IDP can interact with apo-*Sj*PPase (lanes 1 and 7) and the Mg^2+^-*Sj*PPase complex (lanes 6 and 8). There were weaknesses in the interaction between GSH and *Sj*PPase (lanes 1 and 3, or lanes 2 and 8).

### Enzymatic Activity of Recombinant *Sj*PPase

**FIGURE 4:**
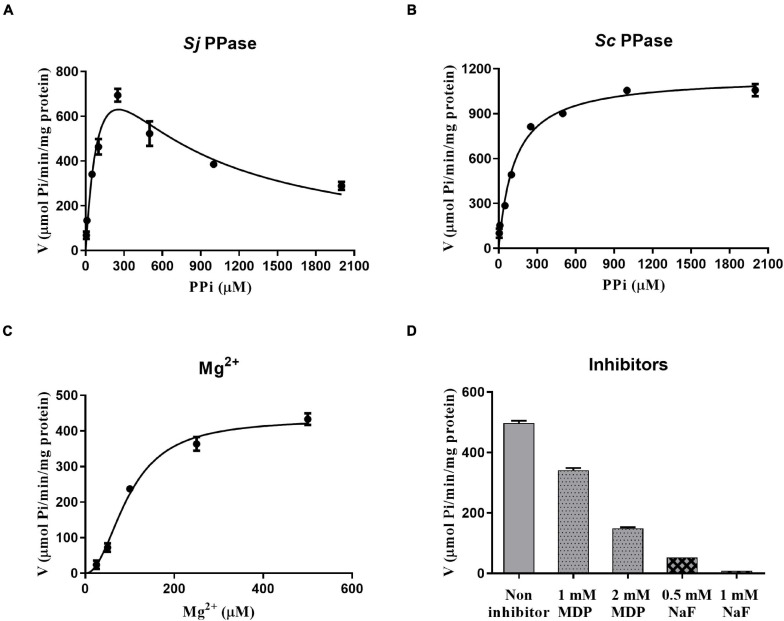
The rates of enzymic reaction under different conditions. Panels **(A–D)** represent various concentrations of inorganic pyrophosphates (PPi), magnesium ions (Mg^2+^), sodium fluoride (NaF), and methylene diphosphonic acid (MDP), respectively. The magnesium ion is not only a cofactor but also a partial component of the substrate. Inorganic pyrophosphates, together with magnesium ions, become the substrate of the enzyme PPase. NaF and MDP are the inhibitors against PPase.

The PPase activity was analyzed using a Pi Per^TM^ Phosphate assay Kit (Molecular Probes, Eugene, OR, United States). We investigated the influence of PPi on *Sj*PPase and *Sc*PPase activity (Figures 4A,B). In contrast to *Sc*PPase (higher PPi concentration and higher activity), *Sj*PPase presents a maximum activity at a concentration of 250 μM PPi. Inhibition of enzyme activity was observed when the concentration of PPi was over 250 μM. In addition, the effect of various concentrations of Mg^2+^ on the *Sj*PPase activity was determined (Figure 4C). The enzyme activity of *Sj*PPase increases with the concentration of Mg^2+^ from 25 to 500 μM. Subsequently, it was observed that the inhibitors NaF and MDP inhibited the enzymatic activity of *Sj*PPase (Figure 4D).

### Structure of *Sj*PPase

**FIGURE 5:**
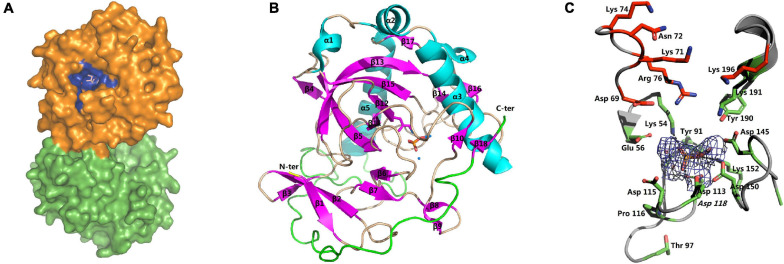
Overall structure and active site of *Sj*PPase. **(A)** The centrosymmetric dimer of *Sj*PPase is seen on the surface. The blue site represents the active site of the enzyme formed by the conserved residues. **(B)** The structure of the *Sj*PPase monomer (β: beta-sheet, α: alpha-helix, θ: 3_10_ alpha-helix, N-ter: amino-terminal, C-ter: carboxyl-terminal). The β-sheets are in pink, and α-helices are in cyan. **(C)** The *Sj*PPase active site. A stereoview of the active site in the *Sj*PPase. The side chain of conserved residues is shown as a green stick. The 2Fo-Fc electron density map covering the Pi and cobalt ions is shown as a mesh at the 2.1 sigma. Hydrogen bonds are shown in dashed lines. The carbon skeleton of residues in red forms the product releasing channel.

The structures of apo-*Sj*PPase (4QMB) and *Sj*PPase-Pi (4QLZ) were similar. Overall, the root mean square deviation (RMSD) between the two structures was 0.454 Å (Supplementary Table 4). The two structures consist of two molecules in the asymmetric unit and also form a homodimer (Figure 5A). The two subunits in one asymmetric unit contain residues 1–284 and 1–283 out of a total of 287 residues, respectively. Superimposition of the two subunits leads to RMSDs of 0.29 Å (4QLZ) and 0.12 Å (4QMB). The monomers (Figure 5B) were arranged in a compact globular shape consisting of several β-sheets and α-helixes, one 3_10_-helix, and some loops. The conservative residues of active sites were located at the core of the structure.

The apo-*Sj*PPase and *Sj*PPase-Pi had some differences. The main distinction is that there were ligands (four cobalt ions and one phosphate) in one *Sj*PPase-Pi unit but not in apo-*Sj*PPase. By alignment of secondary structure, it becomes apparent that changes occur in residues 132–157 involving four conserved residues (Gly139, Asp145, Asp150, and Lys152) (Figure 1). Due to the interaction between these ligands and *Sj*PPase, the β_132–145_-turn_146–147_-β_148–157_ of 4QMB becomes β_132–141_-loop_142_-β_143–145_-turn_146–147_-β_148–149_-loop_150–151_-β_152–157_ of 4QLZ (Figure 1).

**FIGURE 6:**
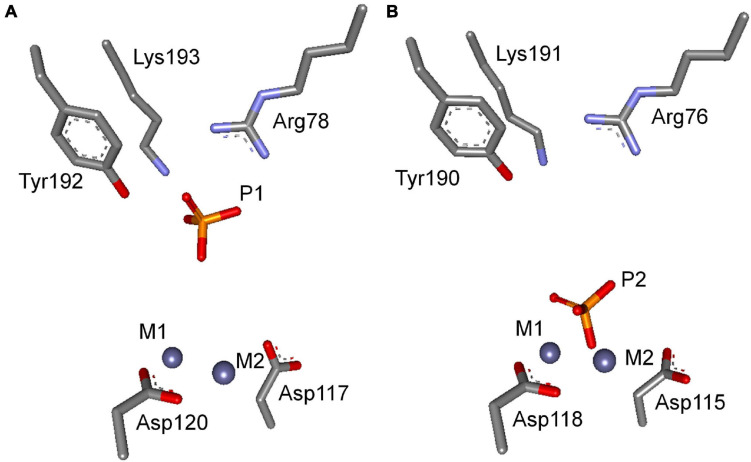
The comparison of F intermediate at the enzymatic site between *Sj*PPase and ScPPase. **(A)** the F intermediate of *Sc*PPase; **(B)** the F intermediate of *Sj*PPase. The F intermediate refers to the state where one Pi remains in the active site, and another Pi has been released. However, the structural comparison suggests that the F state of *Sj*PPase and *Sc*PPase have a distinct discrepancy. The remaining Pi of the F intermediate was P2 (down) in *Sj*PPase but P1 (up) in *Sc*PPase.

The active site of *Sj*PPase is shown in Figure 5C. The overall shape and size of the active site were very similar to those of *Sc*PPases (Tuominen et al., 1998). Ligands including magnesium acted as metal cofactors, and phosphate hydrolyzed products were incorporated in this structure. These ligand interactions were mainly derived from the hydrogen donors/receptors and coordination bond atoms. From the perspective of the six-state mechanism of PPase (Oksanen et al., 2007), the structure of the *Sj*PPase-Pi complex is in the F intermediate catalytic cycle, which is the stage at which one Pi is released after PPi hydrolysis. However, the F intermediates differ in the two structures (4QLZ and 2IHP). Therefore, the Pi in the *Sc*PPase-Pi complex structure is at the P1 site (Figure 6A), and the Pi in the *Sj*PPase-Pi complex structure is at the P2 site (Figure 6B).

### Docking of PPi and MDP Into the *Sj*PPase Active Site

**FIGURE 7:**
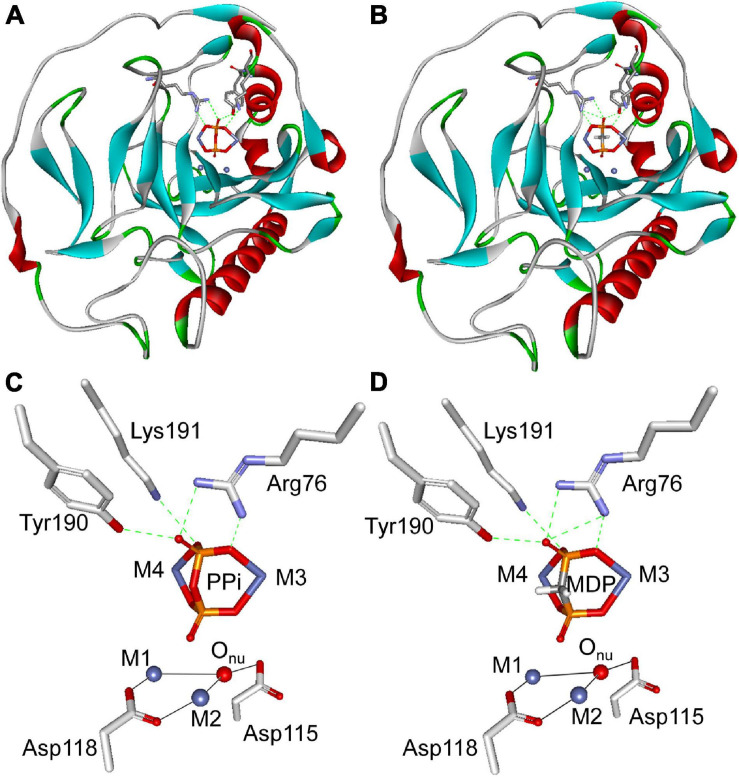
Molecular docking analysis of PPi and MDP with *Sj*PPase. The protein is shown as a ribbon diagram, color-coded according to its secondary structure. Selected residues are displayed as gray backbones. PPi and MDP are colored according to atom type (phosphorous atoms are colored in orange, oxygen atoms in red, carbon atoms in gray, and Co ions in royal blue). Metals (M1 and M2) are represented by royal blue spheres, coordination bonds by black solid lines, and hydrogen bonds by green dashed lines. **(A)** Illustration of PPi docked into the active site of *Sj*PPase. **(B)** Illustration of MDP docked into the active site of *Sj*PPase. **(C)** Stereoview of PPi docked into the active site of *Sj*PPase. **(D)** Stereoview of MDP docked into the active site of *Sj*PPase.

Molecular studies were conducted using the CDOCKER program in the Discovery Studio 2.1 software package to provide information on the competitive inhibition of MDP on *Sj*PPase. The X-ray crystal structure of *Sj*PPase was used for docking calculation. The representative positions of MDP and PPi considered in this work are shown in Figure 7. Generally, MDP can mimic PPi precisely to interact with *Sj*PPase at the same binding site. In addition, MDP exhibited a similar binding mode compared to PPi (Figures 7A,B), and the same was also observed in yeast PPase (Oksanen et al., 2007). A few important hydrogen bonds are involved in the interactions. The docking models illustrated that PPi might form four hydrogen bond interactions with three key residues in the active site of *Sj*PPase (Arg76, Tyr190, and Lys191) (Figure 7C). In the case of MDP, except for the four hydrogen bonds, an extra hydrogen bond was formed between the carbonyl oxygen atom and Arg76, as shown in Figure 7D. Moreover, MDP has a lower CDOCKER energy than PPi (MDP: –186.23 kcal/mol; PPi: –171.50 kcal/mol). Furthermore, MDP could serve as a perfect PPi mimic; it is not surprising that MDP exhibits a significantly competitive inhibition effect on *Sj*PPase.

**FIGURE 8:**
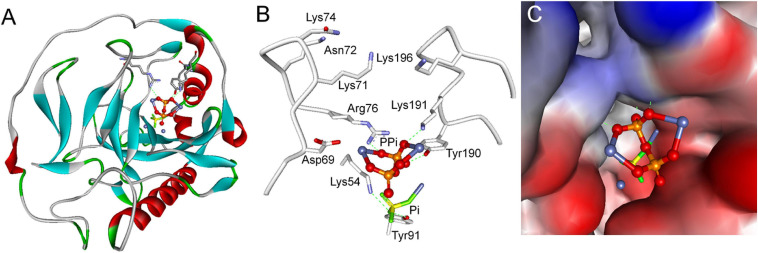
Simulation models of substrate inhibition of *Sj*PPase by PPi. **(A)** Overall structure of the *Sj*PPase-Pi complex with PPi docked into its active site. The protein is shown as a ribbon diagram, color-coded according to its secondary structure. Metals (M1 and M2) are represented by royal blue spheres. **(B)** Stereoview of PPi in the product release channel of *Sj*PPase. Selected residues are represented by gray backbones and hydrogen bonds by green dashed lines. **(C)** Surface view of PPi in the product release channel of *Sj*PPase. The protein is shown in surface view, color-coded according to its interpolated charge.

Furthermore, docking simulations were performed using the CDOCKER program within Discovery Studio 2.1 software package to deepen our understanding of substrate inhibition of *Sj*PPase by PPi. The simulation was carried out using the X-ray crystal structure of *Sj*PPase. Results obtained show that PPi bound just at the entrance of the product release channel (Figure 8A), which was formed by Lys54, Lys71, Asn72, Lys74, Arg76, Tyr190, Lys191, and Lys196 (Figure 8B). PPi might form three hydrogen bond interactions with three key residues in the product release channel (Arg76, Tyr190, and Lys191) (Figure 8B). Product (Pi) was supposed to be released through the flexible positively charged channel (Oksanen et al., 2007), which could provide a low-energy pathway out by passing Pi along the chain of lysines (Oksanen et al., 2007). As shown in the simulation models, PPi blocked the exit of Pi and cut off its release pathway (Figure 8C), which might explain the mechanism of action of the substrate inhibition of *Sj*PPase.

## Discussion

Previous phylogenetic tree results (Sivula et al., 1999) suggested that soluble PPases (sPPase) are grouped into three subfamilies, i.e., plant PPases, prokaryotic PPases, and animal and fungal PPases. The alignment results and phylogenetic analysis (data not shown) indicate that inorganic pyrophosphatase from *S. japonicum* belongs to the animal PPases of sPPase family I. The alignment of protein sequences revealed that 15 residues are conserved in some family I sPPase sequences (Figure 1). Although these conserved residues are separated in sequence, they participated in forming the active site (Tuominen et al., 1998; Avaeva, 2000; Chao et al., 2006) for PPi hydrolysis from a structural standpoint (Figure 5C). Most eukaryotic PPases are homodimers; crystal structure results suggest that *Sj*PPase is also a homodimer (Figure 5A). Interestingly, the activity of human PPase (HuPPase) depends on self-assemblies by interdimeric contacts of Arg52–Asp281 (Hu et al., 2020). The Arg52/Asp281 of HuPPase corresponds to Arg49/Glu278 of *Sj*PPase in multiple alignments (data not shown). The effect of the Glu278 of *Sj*PPase on its self-assembly and activity is subject to further studies.

To better understand the role of PPase in *Schistosoma*, we analyzed the metabolic pathway of substrate PPi by enzymatic reaction analysis, a literature search, and sequence alignment. iPPi was primarily generated by pyrophosphorolysis (Figure 2); the generated iPPi can be quickly hydrolyzed by sPPase. However, sPPase hydrolysis is not the only approach to reduce iPPi levels (Serrano-Bueno et al., 2013). Other ways, such as transshipment by the PPi transporter (ANKH) (Ryan, 2001; Netter et al., 2004), hydrolysis by alkaline phosphatase (AP) (Araujo-Montoya et al., 2011), and utilization by some enzymes, can also reduce iPPi concentration. ANKH genes are found in most vertebrate tissues (Ho et al., 2000; Nurnberg et al., 2001; Foster et al., 2012); however, no ANKH gene has been found in the genome of *Schistosoma* by sequence alignment. Enzymes that utilize PPi in *Schistosoma* mainly include some transferases (Yuan et al., 1990; Yang et al., 2007; Ge et al., 2011). In relation to these results (Figure 2), the iPPi generated by pyrophosphorolysis in *Schistosoma* is regulated by sPPase and AP together with transferases that utilize PPi.

Soluble inorganic pyrophosphatase is one of the cellular tools that reduce iPPi concentration in *Schistosoma*. As previously analyzed and exemplified (Islam et al., 2003; Ko et al., 2007), sPPase can facilitate the growth and development of organisms by hydrolyzing iPPi. Furthermore, sPPase can also eliminate the rapidly generated PPi to prevent cell injury or even death that might result from the accumulation of iPPi under certain conditions (George et al., 2010; Serrano-Bueno et al., 2013). Hence, the efficacy of sPPase catalytic activity is significant for performing its functions. The enzyme assay results suggested that *Sj*PPase possesses PPi hydrolysis activity in a dose-dependent manner with Mg^2+^ (Figure 4C).

In addition, *Sj*PPase activity that hydrolyzes PPi can be inhibited by different inhibitors with different mechanisms, including excess PPi, NaF, and MDP—their influence on PPase enzyme activity is shown in Figures 4A,D. The inhibition of PPase by NaF resulted from the substitution of a water molecule by NaF in the active site (Heikinheimo et al., 2001; Pohjanjoki et al., 2001). The force of interaction is stronger between Mg^2+^ and NaF than between Mg^2+^ and water molecules. Therefore, NaF substitutes water molecules in the active site and blocks water molecules from attacking pyrophosphate bonds. Consequently, the hydrolysis of PPi is inhibited by NaF. MDP is a substrate analog (Zyryanov et al., 2005) and substrate competitive inhibitor (Figure 7). Therefore, the inhibition of PPase by MDP was caused by the competitive binding of MDP and PPi to the active site (Figure 7; Gordon-Weeks et al., 1999). Due to high substrate similarity (Figures 7C,D), MDP easily binds to the PPase active site. PPase cannot hydrolyze MDP; therefore, the catalytic cycle is aborted at the inhibition-binding state. As a result, the hydrolysis of PPi is inhibited. Although the two inhibitors (NaF and MDP) show different mechanisms, their active sites are adjacent. This may give us a clue to pursue a novel inhibitor with high efficiency and selectivity.

The effect of substrate PPi on *Sj*PPase suggests that excess PPi can inhibit enzyme activity (Figure 4A and Supplementary Figure 1). Due to the increasing concentration of Pi with the hydrolysis of PPi, two variable factors (the concentrations of PPi and Pi) are present in the reaction. Therefore, this leads to the question of which factor is directly responsible for inhibition. A Pi-time curve of the reaction (Supplementary Figure 1) revealed that PPi is the causative factor. The detailed explanation involves three arguments: (1) excess PPi inhibits *Sj*PPase activity at the initial stage of the reaction (the concentration of Pi is extremely low); (2) the inhibition of *Sj*PPase activity was not enhanced with an increase in Pi (Supplementary Figure 1); and (3) the inhibition of *Sj*PPase activity was enhanced with increased PPi (Figure 4A and Supplementary Figure 1). Therefore, inhibition is caused by substrate PPi, and it is termed substrate inhibition.

Soluble inorganic pyrophosphatase is not just one of the cellular tools that reduce iPPi concentration in *Schistosoma*. Substrate inhibition is often regarded as a non-physiological phenomenon; however, previous reports have accumulated evidence proving that it is a biologically relevant regulatory mechanism (Reed et al., 2010). Substrate PPi is an energy-rich compound; therefore, with reference to energy utilization, substrate inhibition of *Sj*PPase may reduce reserved energy wastage in PPi. In addition, the abundant PPi generated from pyrophosphorolysis reactions can participate in the reactions catalyzed by transferases utilizing PPi (Figure 2; Yuan et al., 1992). Furthermore, PPi hydrolysis is accompanied by the release of heat (da-Silva et al., 2004); as such, a situation whereby abundant PPi is rapidly generated and completely hydrolyzed in a cell might cause thermal injury, which could lead to the eventual death of the cell. However, the situation may not be completely hopeless; as mentioned earlier, cells containing ANKH, such as human cells (Figure 2A), do not die due to the heat produced by PPi hydrolysis because the PPi generated is pumped out of the cell by ANKH. The question is on what happens to *Schistosoma* cells without ANKH (Figure 2B). Evidently, *Schistosoma* cells do not die due to overheating caused by excessive PPi hydrolysis for the substrate inhibition of PPase. Furthermore, there is limited acceleration in the rate of macromolecular biosynthesis. Therefore, substrate inhibition of *Sj*PPase is physiologically significant in organisms without ANKH, such as *Schistosoma*. Hence, substrate inhibition of *Sj*PPase not only allows complete energy utilization but also helps prevent heat stress that may lead to cell injury and also regulates the rate of macromolecular biosynthesis.

Substrate inhibition of *Sj*PPase is of great importance to *Schistosoma*, but its mechanism is still unknown. Therefore, the occurrence of substrate inhibition is quite interesting, albeit being problematic.

The inhibition phenomenon arising from excess PPi has been previously reported for PPase from the photosynthetic bacterium *Rhodospirillum rubrum* (Klemme and Gest, 1971); however, the mechanism remains unclear. A structural comparison method coupled with site-directed mutagenesis successfully identified the key residues related to substrate inhibition of betaine aldehyde dehydrogenase in *Staphylococcus aureus* (Chen C. et al., 2014). Using a similar method, we found that *Sj*PPase and *Sc*PPase ligand and cofactor binding sites were identical. Unfortunately, we were unable to identify the key residues contributing to substrate inhibition of *Sj*PPase using this method.

Nevertheless, two exciting studies on PPi hydrolysis mechanisms by PPase (Heikinheimo et al., 2001; Oksanen et al., 2007) reported that hydrolysis undergoes a six-state mechanism in catalytic cycles. For the F intermediate (one of the six states), only one Pi binds to the active site, and this has ample room to accommodate at least three Pi (Oksanen et al., 2007). Therefore, the intermediate has enough space to accommodate another PPi. Hence, we proposed a substrate-inhibition hypothesis that substrate PPi binds to an F intermediate and blocks the exit of Pi from the active site, thereby causing the inhibition of enzymatic activity.

An *Sj*PPase F intermediate crystal structure was first prepared to verify this hypothesis (Figure 5). The second Pi is located at the bottom of the product release channel in the intermediate (Figure 5C) and leaves the *Sj*PPase active site momentarily.

It is important to have a good understanding of substrate inhibition regardless of whether PPi binds with *Sj*PPase at the F intermediate and blocks Pi exit from the *Sj*PPase active site. Therefore, we realized that the docking of PPi into the active site of the *Sj*PPase F intermediate allows PPi to bind with the *Sj*PPase F intermediate and block Pi exit (Figure 8).

## Conclusion

In summary, *Sj*PPase is a vehicle for PPi hydrolysis, and its hydrolysis activity toward PPi could be inhibited not only by NaF and MDP but also by excessive substrate PPi (substrate inhibition). Furthermore, the structure of the *Sj*PPase F intermediate was solved at 2.3 Å. In addition, excess PPi blocked the exit of Pi by binding with enzymes in the docking experiment, indicating that substrate inhibition may be caused by excess PPi attacking the *Sj*PPase F intermediate of the catalytic cycle. Lastly, our results provide novel insight into the mechanism of substrate inhibition.

## Data Availability Statement

The datasets presented in this study can be found in online repositories. The names of the repository/repositories and accession number(s) can be found in the article/Supplementary Material.

## Author Contributions

Q-FW, Y-DL, and J-HC conceived and designed the experiments. Q-FW, W-SW, S-BC, and BX performed the experiments. Q-FW, W-SW, Y-DL, and J-HC analyzed the data. S-BC and BX contributed the reagents, materials, and analysis tools. Q-FW, Y-DL, and J-HC wrote the manuscript. All authors contributed to the article and approved the submitted version.

## Conflict of Interest

The authors declare that the research was conducted in the absence of any commercial or financial relationships that could be construed as a potential conflict of interest.

## Publisher’s Note

All claims expressed in this article are solely those of the authors and do not necessarily represent those of their affiliated organizations, or those of the publisher, the editors and the reviewers. Any product that may be evaluated in this article, or claim that may be made by its manufacturer, is not guaranteed or endorsed by the publisher.

## Footnotes

1
http://www.ebi.ac.uk/Tools/msa/muscle/

2
http://espript.ibcp.fr/ESPript/cgi-bin/ESPript.cgi

3
http://www.ebi.ac.uk/thornton-srv/databases/enzymes/

4
https://www.ncbi.nlm.nih.gov/protein

5
http://www.rcsb.org/

## Supplementary Material

Supplementary Figure 1 The Pi-time curve at 250 and 2000 μM PPi.

Supplementary Table 1 The enzymic reaction conditions of *Sj*PPase.

Supplementary Table 2 The conditions of crystals of *Sj*PPase.

Supplementary Table 3 Protein molecules of human and *Schistosoma japonicum* in PPi metablic pathway.

Supplementary Table 4 The structural alignment of the SjPPase (4QLZ and 4QMB) and ScPPase (1E9G).

Supplementary File 1 The sequences of progressive ankylosis protein homology that were downloaded from UniProtKB.

## References

[B1] Adams P. D. Afonine P. V. Bunkoczi G. Chen V. B. Davis I. W. Echols N. (2010). PHENIX: a comprehensive Python-based system for macromolecular structure solution. *Acta Crystallogr. Biol. Crystallogr.* 66 213–221. 10.1107/S0907444909052925 20124702 PMC2815670

[B2] Araujo-Montoya B. O. Rofatto H. K. Tararam C. A. Farias L. P. Oliveira K. C. Verjovski-Almeida S. (2011). *Schistosoma mansoni*: molecular characterization of alkaline phosphatase and expression patterns across life cycle stages. *Exp. Parasitol.* 129 284–291. 10.1016/j.exppara.2011.07.008 21784070

[B3] Avaeva S. M. (2000). Active site interactions in oligomeric structures of inorganic pyrophosphatases. *Biochemistry (Mosc)* 65 361–372. 10739480

[B4] Avila C. Kornilayev B. A. Blagg B. S. (2006). Development and optimization of a useful assay for determining Hsp90’s inherent ATPase activity. *Bioorg. Med. Chem.* 14 1134–1142. 10.1016/j.bmc.2005.09.027 16213144

[B5] Benini S. Wilson K. (2011). Structure of the *Mycobacterium tuberculosis* soluble inorganic pyrophosphatase Rv3628 at pH 7.0. *Acta Crystallogr. Sect. F Struct. Biol. Cryst. Commun.* 67 866–870. 10.1107/S1744309111023323 21821883 PMC3151116

[B6] Chao T. C. Huang H. Tsai J. Y. Huang C. Y. Sun Y. J. (2006). Kinetic and structural properties of inorganic pyrophosphatase from the pathogenic bacterium *Helicobacter pylori*. *Proteins* 65 670–680. 10.1002/prot.21093 16988955

[B7] Chen C. Joo J. C. Brown G. Stolnikova E. Halavaty A. S. Savchenko A. (2014). Structure-based mutational studies of substrate inhibition of betaine aldehyde dehydrogenase BetB from *Staphylococcus aureus*. *Appl. Environ. Microbiol.* 80 3992–4002. 10.1128/AEM.00215-14 24747910 PMC4054205

[B8] Chen J. H. Zhang T. Ju C. Xu B. Lu Y. Mo X. J. (2014). An integrated immunoproteomics and bioinformatics approach for the analysis of *Schistosoma japonicum* tegument proteins. *J. Proteomics* 98 289–299. 10.1016/j.jprot.2014.01.010 24448400

[B9] Costa E. P. Campos E. de Andrade C. P. Facanha A. R. Saramago L. Masuda A. (2012). Partial characterization of an atypical family I inorganic pyrophosphatase from cattle tick *Rhipicephalus (Boophilus) microplus*. *Vet. Parasitol.* 184 238–247. 10.1016/j.vetpar.2011.09.005 22001703

[B10] da-Silva W. S. Bomfim F. M. Galina A. de Meis L. (2004). Heat of PPi hydrolysis varies depending on the enzyme used. Yeast and corn vacuolar pyrophosphatase. *J. Biol. Chem.* 279 45613–45617. 10.1074/jbc.M408866200 15322117

[B11] Edgar R. C. (2004). MUSCLE: multiple sequence alignment with high accuracy and high throughput. *Nucleic Acids Res.* 32 1792–1797. 10.1093/nar/gkh340 15034147 PMC390337

[B12] Emsley P. Cowtan K. (2004). Coot: model-building tools for molecular graphics. *Acta Crystallogr. Biol. Crystallogr.* 60 2126–2132. 10.1107/S0907444904019158 15572765

[B13] Espiau B. Lemercier G. Ambit A. Bringaud F. Merlin G. Baltz T. (2006). A soluble pyrophosphatase, a key enzyme for polyphosphate metabolism in *Leishmania*. *J. Biol. Chem.* 281 1516–1523. 10.1074/jbc.M506947200 16291745

[B14] Foster B. L. Nagatomo K. J. Nociti F. H. Fong H. Dunn D. Tran A. B. (2012). Central role of pyrophosphate in acellular cementum formation. *PLoS One* 7:e38393. 10.1371/journal.pone.0038393 22675556 PMC3366957

[B15] Ge J. Chen H. G. Hu W. (2011). Bioinformatics analysis of nicotinamide phosphoribosyl transferase of *Schistosoma japonicum*. *Chin. J. Schisto. Control* 23 292–295. 22164495

[B16] George G. M. van der Merwe M. J. Nunes-Nesi A. Bauer R. Fernie A. R. Kossmann J. (2010). Virus-induced gene silencing of plastidial soluble inorganic pyrophosphatase impairs essential leaf anabolic pathways and reduces drought stress tolerance in *Nicotiana benthamiana*. *Plant Physiol.* 154 55–66. 10.1104/pp.110.157776 20605913 PMC2938153

[B17] Gordon-Weeks R. Parmar S. Davies T. G. Leigh R. A. (1999). Structural aspects of the effectiveness of bisphosphonates as competitive inhibitors of the plant vacuolar proton-pumping pyrophosphatase. *Biochem. J.* 337(Pt 3) 373–377. 9895279 PMC1219987

[B18] Han Z. G. Brindley P. J. Wang S. Y. Chen Z. (2009). *Schistosoma* genomics: new perspectives on schistosome biology and host-parasite interaction. *Annu. Rev. Genomics Hum. Genet.* 10 211–240. 10.1146/annurev-genom-082908-150036 19630560

[B19] Heikinheimo P. Tuominen V. Ahonen A. K. Teplyakov A. Cooperman B. S. Baykov A. A. (2001). Toward a quantum-mechanical description of metal-assisted phosphoryl transfer in pyrophosphatase. *Proc. Natl. Acad. Sci. U.S.A.* 98 3121–3126. 10.1073/pnas.061612498 11248042 PMC30617

[B20] Ho A. M. Johnson M. D. Kingsley D. M. (2000). Role of the mouse ank gene in control of tissue calcification and arthritis. *Science* 289 26 5–270. 10894769 10.1126/science.289.5477.265

[B21] Hoelzle K. Peter S. Sidler M. Kramer M. M. Wittenbrink M. M. Felder K. M. (2010). Inorganic pyrophosphatase in uncultivable hemotrophic mycoplasmas: identification and properties of the enzyme from *Mycoplasma suis*. *BMC Microbiol.* 10:194. 10.1186/1471-2180-10-194 20646294 PMC2916918

[B22] Hu F. Huang Z. Zheng S. Wu Q. Chen Y. Lin H. (2020). Structural and biochemical characterization of inorganic pyrophosphatase from Homo sapiens. *Biochem. Biophys. Res. Commun.* 533 1115–1121. 10.1016/j.bbrc.2020.09.139 33036755

[B23] Islam M. K. Miyoshi T. Kasuga-Aoki H. Isobe T. Arakawa T. Matsumoto Y. (2003). Inorganic pyrophosphatase in the roundworm *Ascaris* and its role in the development and molting process of the larval stage parasites. *Eur. J. Biochem.* 270 2814–2826. 12823552 10.1046/j.1432-1033.2003.03658.x

[B24] Jamwal A. Round A. R. Bannwarth L. Venien-Bryan C. Belrhali H. Yogavel M. (2015). Structural and Functional highlights of vacuolar soluble protein 1 from pathogen *Trypanosoma brucei* brucei. *J. Biol. Chem.* 290 30498–30513. 10.1074/jbc.M115.674176 26494625 PMC4683271

[B25] Klemme J. H. Gest H. (1971). Regulatory properties of an inorganic pyrophosphatase from the photosynthic bacterium *Rhodospirillum rubrum*. *Proc. Natl. Acad. Sci. U.S.A.* 68 721–725. 4396317 10.1073/pnas.68.4.721 PMC389028

[B26] Ko K. M. Lee W. Yu J. R. Ahnn J. (2007). PYP-1, inorganic pyrophosphatase, is required for larval development and intestinal function in *C. elegans*. *FEBS Lett.* 581 5445–5453. 10.1016/j.febslet.2007.10.047 17981157

[B27] Liu F. Lu J. Hu W. Wang S. Y. Cui S. J. Chi M. (2006). New perspectives on host-parasite interplay by comparative transcriptomic and proteomic analyses of *Schistosoma japonicum*. *PLoS Pathog.* 2:e29. 10.1371/journal.ppat.0020029 16617374 PMC1435792

[B28] Netter P. Bardin T. Bianchi A. Richette P. Loeuille D. (2004). The ANKH gene and familial calcium pyrophosphate dihydrate deposition disease. *Joint Bone Spine* 71 365–368. 10.1016/j.jbspin.2004.01.011 15474385

[B29] Nurnberg P. Thiele H. Chandler D. Hohne W. Cunningham M. L. Ritter H. (2001). Heterozygous mutations in ANKH, the human ortholog of the mouse progressive ankylosis gene, result in craniometaphyseal dysplasia. *Nat. Genet.* 28 37–41. 10.1038/88236 11326272

[B30] Oksanen E. Ahonen A. K. Tuominen H. Tuominen V. Lahti R. Goldman A. (2007). A complete structural description of the catalytic cycle of yeast pyrophosphatase. *Biochemistry* 46 1228–1239. 10.1021/bi0619977 17260952

[B31] Otwinowski Z. Minor W. (1997). Processing of X-ray diffraction data collected in oscillation mode. *Methods Enzymol.* 276 307–326. 10.1016/S0076-6879(97)76066-X 27754618

[B32] Pace D. A. Fang J. Cintron R. Docampo M. D. Moreno S. N. (2011). Overexpression of a cytosolic pyrophosphatase (TgPPase) reveals a regulatory role of PP(i) in glycolysis for *Toxoplasma gondii*. *Biochem. J.* 440 229–240. 10.1042/BJ20110641 21831041 PMC4874478

[B33] Pohjanjoki P. Fabrichniy I. P. Kasho V. N. Cooperman B. S. Goldman A. Baykov A. A. (2001). Probing essential water in yeast pyrophosphatase by directed mutagenesis and fluoride inhibition measurements. *J. Biol. Chem.* 276 434–441. 10.1074/jbc.M007360200 11031269

[B34] Reed M. C. Lieb A. Nijhout H. F. (2010). The biological significance of substrate inhibition: a mechanism with diverse functions. *Bioessays* 32 422–429. 10.1002/bies.200900167 20414900

[B35] Robert X. Gouet P. (2014). Deciphering key features in protein structures with the new ENDscript server. *Nucleic Acids Res.* 42 W320–W324. 10.1093/nar/gku316 24753421 PMC4086106

[B36] Ryan L. M. (2001). The ank gene story. *Arthritis Res.* 3 77–79. 10.1186/ar143 11178113 PMC128882

[B37] Serrano-Bueno G. Hernandez A. Lopez-Lluch G. Perez-Castineira J. R. Navas P. Serrano A. (2013). Inorganic pyrophosphatase defects lead to cell cycle arrest and autophagic cell death through NAD+ depletion in fermenting yeast. *J. Biol. Chem.* 288 13082–13092. 10.1074/jbc.M112.439349 23479727 PMC3642350

[B38] Sitnik T. S. Avaeva S. M. (2007). Binding of substrate at the effector site of pyrophosphatase increases the rate of its hydrolysis at the active site. *Biochemistry (Mosc)* 72 68–76. 17309439 10.1134/s0006297907010087

[B39] Sitnik T. S. Vainonen J. P. Rodina E. V. Nazarova T. I. Kurilova S. A. Vorobyeva N. N. (2003). Effectory site in *Escherichia coli* inorganic pyrophosphatase is revealed upon mutation at the intertrimeric interface. *IUBMB Life* 55 37–41. 10.1080/1521654031000072139 12716061

[B40] Sivula T. Salminen A. Parfenyev A. N. Pohjanjoki P. Goldman A. Cooperman B. S. (1999). Evolutionary aspects of inorganic pyrophosphatase. *FEBS Lett.* 454 75–80. 10413099 10.1016/s0014-5793(99)00779-6

[B41] Stockbridge R. B. Wolfenden R. (2011). Enhancement of the rate of pyrophosphate hydrolysis by nonenzymatic catalysts and by inorganic pyrophosphatase. *J. Biol. Chem.* 286 18538–18546. 10.1074/jbc.M110.214510 21460215 PMC3099670

[B42] Tuominen V. Heikinheimo P. Kajander T. Torkkel T. Hyytia T. Kapyla J. (1998). The R78K and D117E active-site variants of Saccharomyces cerevisiae soluble inorganic pyrophosphatase: structural studies and mechanistic implications. *J. Mol. Biol.* 284 1565–1580. 10.1006/jmbi.1998.2266 9878371

[B43] Villa-Bellosta R. Rivera-Torres J. Osorio F. G. Acín-Pérez R. Enriquez J. A. López-Otín C. (2013). Defective extracellular pyrophosphate metabolism promotes vascular calcification in a mouse model of hutchinson-gilford progeria syndrome that is ameliorated on pyrophosphate treatment. *Circulation* 127 2442–2451. 10.1161/CIRCULATIONAHA.112.000571 23690466

[B44] Villa-Bellosta R. Wang X. Millan J. L. Dubyak G. R. O’Neill W. C. (2011). Extracellular pyrophosphate metabolism and calcification in vascular smooth muscle. *Am. J. Physiol. Heart Circ. Physiol.* 301 H61–H68. 10.1152/ajpheart.01020.2010 21490328 PMC3129914

[B45] Yang Z. Hu W. Su J. Ma L. Li Y. X. Feng Z. (2007). Bioinformatic identification and analysis of *Schistosoma japonicum* adenine phosphoribosyltransferase. *J. South Med. Univ.* 27 27 2–275. 17425969

[B46] Yi Y. J. Sutovsky M. Kennedy C. Sutovsky P. (2012). Identification of the inorganic pyrophosphate metabolizing, ATP substituting pathway in mammalian spermatozoa. *PLoS One* 7:e34524. 10.1371/journal.pone.0034524 22485177 PMC3317647

[B47] Yuan L. Craig S. P. McKerrow J. H. Wang C. C. (1990). The hypoxanthine-guanine phosphoribosyltransferase of *Schistosoma mansoni*. Further characterization and gene expression in *Escherichia coli*. *J. Biol. Chem.* 265 13528–13532. 2199439

[B48] Yuan L. Craig S. P. McKerrow J. H. Wang C. C. (1992). Steady-state kinetics of the schistosomal hypoxanthine-guanine phosphoribosyltransferase. *Biochemistry* 31 806–810. 1731938 10.1021/bi00118a024

[B49] Zyryanov A. B. Lahti R. Baykov A. A. (2005). Inhibition of family II pyrophosphatases by analogs of pyrophosphate and phosphate. *Biochemistry (Mosc)* 70 908–912. 16212547 10.1007/s10541-005-0201-5

