# Supplementary material for: Crystal Structure of Inorganic Pyrophosphatase From Schistosoma japonicum Reveals the Mechanism of Chemicals and Substrate Inhibition
Source: Front Cell Dev Biol. 2021 Aug 11;9:712328. doi: 10.3389/fcell.2021.712328 (PMC8386120; doi:10.3389/fcell.2021.712328)
Supplement: Supplementary Table 1 — The enzymic reaction conditions of SjPPase. [file Table_1.docx]

**Supplementary table 1. The enzymatic reaction conditions of *Sj*PPase**

-------------------------------------------------------------------------------------------------------

50μl 50μl

----------------------------------------------------------------------

Group *Sj*PPase (μg) Mg^2+^ (μM) PPi (μM) F^-^ MDP

-------------------------------------------------------------------------------------------------------

A test 2 VC_A_ 200 - -

A control - VC_A_ 200 - -

B test 2 250 VC_B_ - -

B control - 250 VC_B_ - -

C test 2 250 100 VC_C_ -

C control - 250 100 VC_C_ -

D test 2 250 100 - VC_D_

D control - 250 100 - VC_D_

-------------------------------------------------------------------------------------------------------

Each of group contains two reactions, test and control. Each of reaction is a volumn of 100μl reaction system. The enzyme of *Sj*PPase and magnesium ions are mixed with a volumn of 50μl. Inorganic pyrophosphate and fluorinion or MDP are mixed with a volumn of 50μl. VC is short for “various concentration”. VC_A_ represents a series of concentration of magnesium ions, which are 500μM, 250μM, 100μM, 50μM and 25μM, respectively. Various concentration of PPi represented by VC_B_ are 2000μM, 1000μM, 500μM, 250μM, 100μM, 50μM, 10μM and 5μM, respectively. Various concentration of NaF represented by VC_C_ are 1mM, 0.5mM and 0.5mM, respectively. Various concentration of MDP represented by VC_D_ are 2mM and 1mM, respectively.
