# Supplementary material for: Crystal Structure of Inorganic Pyrophosphatase From Schistosoma japonicum Reveals the Mechanism of Chemicals and Substrate Inhibition
Source: Front Cell Dev Biol. 2021 Aug 11;9:712328. doi: 10.3389/fcell.2021.712328 (PMC8386120; doi:10.3389/fcell.2021.712328)
Supplement: Supplementary Table 2 — The conditions of crystals of SjPPase. [file Table_2.docx]

**Supplementary table 2. The conditions of crystals of *Sj*PPase**

-------------------------------------------------------------------------------------------------------

PDB Conditions

-------------------------------------------------------------------------------------------------------

4QLZ 0.1M Tris-HCl pH8.0, 24%PEG 3350, 0.4M MgCl_2_, 0.4M Sodium malonate

4QMB 0.1M Tris-HCl pH 6.8, 2M NH_4_SO_4_, 0.04M MgCl_2_

-------------------------------------------------------------------------------------------------------
