# Supplementary material for: Crystal Structure of Inorganic Pyrophosphatase From Schistosoma japonicum Reveals the Mechanism of Chemicals and Substrate Inhibition
Source: Front Cell Dev Biol. 2021 Aug 11;9:712328. doi: 10.3389/fcell.2021.712328 (PMC8386120; doi:10.3389/fcell.2021.712328)
Supplement: Supplementary Table 4 — The structural alignment of the SjPPase (4QLZ and 4QMB) and ScPPase (1E9G). [file Table_4.docx]

**Supplementary table 4. The structural alignment of the *Sj*PPase (4QLZ and 4QMB) and *Sc*PPase (1E9G)**

-------------------------------------------------------------------------------------------------------

RMSD

-------------------------------------------------------------------------------------------------------

4QLZ and 1E9G 0.615 Å

4QMB and 1E9G 0.727 Å

4QLZ and 4QMB 0.454 Å

-------------------------------------------------------------------------------------------------------
